# Cost-effectiveness of medical interventions to prevent cardiovascular disease in a sub-Saharan African country – the case of Tanzania

**DOI:** 10.1186/1478-7547-5-3

**Published:** 2007-02-22

**Authors:** Bjarne Robberstad, Yusuf Hemed, Ole F Norheim

**Affiliations:** 1 Department of Public Health and Primary Care, University of Bergen, P.o.Box 7804, 5020 Bergen, Norway; 2 MEASURE Evaluation, P.O Box 65243, Dar es Salaam, Tanzania

## Abstract

**Background:**

There is a high and rising prevalence of cardiovascular risk in sub-Saharan Africa, a development typical for countries in epidemiological transition. Contrary to recommendations in treatment guidelines, medical interventions to prevent cardiovascular disease are implemented only on a limited scale in these settings. There is a widespread concern that such treatment is not cost-effective compared to alternative health interventions. The main objectives of this article are therefore to calculate costs-, effects and cost-effectiveness of fourteen medical interventions of primary prevention of cardiovascular disease in Tanzania, including Acetylsalicylic acid, a diuretic drug (Hydrochlorothiazide), a β-blocker (Atenolol), a calcium channel blocker (Nifedepine), a statin (Lovastatin) and various combinations of these.

**Methods:**

Effect sizes were derived from systematic reviews or meta-analyses, and calculated as Disability Adjusted Life Years (DALYs). Data on drug costs were calibrated to a Tanzanian setting. Other recurrent and capital costs were derived from previous studies and reviewed by local experts. Expected lifetime costs and health outcomes were calculated using a life-cycle model. Probabilistic cost-effectiveness analysis was performed using Monte Carlo simulation, and results presented as cost-effectiveness acceptability curves and frontiers. The potential impacts of uncertainty in value laden single parameters were explored in one-way sensitivity analyses.

**Results:**

The incremental cost-effectiveness ratios for the fourteen interventions and four different levels of risk (totally 56 alternative interventions) ranged from about USD 85 per DALY to about USD 4589 per DALY saved. Hydrochlorothiazide as monotherapy is the drug yielding the most favorable cost-effectiveness ratio, although not significantly lower than when it is combined in duo-therapy with Aspirin or a β-blocker, in triple-therapy with Aspirin and a β-blocker, or than Aspirin given as mono-therapy.

**Conclusion:**

Preventive cardiology is not cost-effective for any patient group in this setting until willingness to pay exceeds USD 85 per DALY. At this level of willingness to pay, the optimal intervention is Hydrochlorothiazide to patients with very high cardiovascular risk. As willingness to pay for health increase further, it becomes optimal to provide this treatment also to patients with lower cardiovascular risk, and to substitute to more sophisticated interventions.

## Background

### The epidemiological transition

Cardiovascular diseases (CVD) currently contributes to almost one third of global mortality [1], and has been projected to become the leading cause of global burden of disease by 2020 [2]. In 2001 ischaemeic heart disease represented more than 12% and cerebrovascular diseases almost 10% of the premature deaths globally [3]. The increasing impact from CVD also takes place in developing countries, the so called epidemiological transition [1]. Studies from sub-Saharan Africa (SSA) and Tanzania show that there is a high and rising prevalence of cardiovascular risk in the population [1,4,5]. A survey of the population of Dar es Salaam found an age-adjusted prevalence of about 30% for blood pressure values larger or equal to 140/90 mmHg [5], which leads to increased risk of stroke and coronary heart disease (CHD). The age-adjusted incidence of cardiovascular diseases, like stroke, is furthermore several times higher in Tanzania than in Western Europe [6], probably due to untreated hypertension [5,7].

Cardiologists have discussed whether medical interventions to prevent cardiovascular disease in low-income countries should be given priority, as compared to other interventions competing for funding [8]. Medical interventions are recommended in international as well as national treatment guidelines [9-11], but they are implemented only on a very limited scale in sub-Saharan African countries [11]. While some have argued that pharmaceutical interventions to prevent cardiovascular disease should not be provided due to high costs, others have emphasized that this conclusion is based on an information vacuum [8]. Given the resource situations in many countries in SSA, we share the opinion that particular consideration should be given to the questions of affordability and cost-effectiveness [11], and that more economic evidence as such is useful.

To the best of our knowledge only one study has explored the cost-effectiveness of preventive cardiology in Africa. Murray and colleagues calculated that several medical interventions to lower blood pressure and cholesterol are cost-effective in two African regions [12]. They found that for the region including East-Africa the cost effectiveness of an absolute risk approach varied between 230 and 1010 international dollars per DALY averted depending on the risk level [12]. These figures are taken from webtable 2, and have been multiplied by 10 to adjust for a typing error in the publications (Lancet Department of Error).

When considering health care priorities in Tanzania, the study by Murray and colleagues is useful, but appear insufficient as evidence base for two main reasons. First, the cost-effectiveness ratios are based on aggregated data for a region with large demographic, epidemiological and socio-economic variation. Country specific studies are therefore important, a point also emphasized by the WHO CHOICE team [13]. Second, their study includes only a sub-set of possible medical interventions. A large range of available drug groups and combinations are available and should be considered for implementation. We have explored the relevance of 14 prevention alternatives for Tanzania, which is a typical resource poor sub-Saharan African setting. Similar to the Murray study, we explore the relevance of these pharmaceutical interventions within four different levels of absolute cardiovascular risk, so that the total amount of interventions under consideration is 56.

There are large evidence gaps for preventive cardiology in sub-Saharan Africa [11]. For example, no long-term placebo controlled trials for relevant drugs have been undertaken in Africa. Facing this situation, one option would be to delay our assessment until such evidence is available, but in the light of the dramatic epidemiological development taking place, we think this is too long for African decision makers to wait for economical evidence. The objectives of this paper are therefore to calculate costs-, effects and cost-effectiveness for medical interventions to prevent cardiovascular disease in a low-income country in sub-Saharan Africa using the best available evidence. Our analysis is limited to primary prevention to patients with no history of previous cardiovascular events who are not suffering from other major conditions like HIV-AIDS.

### Description of interventions

We compared 14 alternative medical interventions for preventive cardiology, including the alternative of no treatment. The choice of interventions was based on international treatment recommendations [14], recommendations from a WHO expert meeting [15], Tanzanian guidelines and consultations with local experts. All drugs that are included are off-patent and can be purchased from several producers at relatively low cost. The treatment alternatives are based on five different drugs provided as mono-therapies, duo-therapies, triple-therapies and a therapy combining four different drugs groups. We also included a hypothetical "polypill" in the analysis which remains to be manufactured, but has been described as having "enormous potential in developing countries" [16]. The "polypill" contains a statin, three classes of antihypertensives at half standard dose, folic acid and a low dose of aspirin [17]. An overview of the alternative drugs and drug combinations and the average dosages used in the analysis is given in Table 1.

**Table 1:** The interventions, generic drug names and dosages for the drug combinations considered in the analysis.

**Intervention**	**Generic drug**	**Daily dose**
Aspirin (Asa)	*Acetylsalicylic acid*	75 mg
Diuretic drug (Diu)	*Hydrochlorothiazide*	25 mg
β-blocker (Bet)	*Atenolol*	50 mg
Calcium antagonist (Cab)	*Nifedepine*	40 mg
Statin (Sta)	*Lovastatin*	40 mg
Aspirin + Diuretic drug (AsaDiu)	*Acetylsalicylic acid*	75 mg
	*Hydrochlorothiazide*	25 mg
Aspirin + β-blocker (AsaBet)	*Acetylsalicylic acid*	75 mg
	*Atenolol*	50 mg
Diuretic drug + β-blocker (DiuBet)	*Hydrochlorothiazide*	25 mg
	*Atenolol*	50 mg
Aspirin + Diuretic drug + β-blocker (AsaDiuBet)	*Acetylsalicylic acid*	75 mg
	*Hydrochlorothiazide*	25 mg
	*Atenolol*	50 mg
Aspirin + Diuretic drug + Statin (AsaDiuSta)	*Acetylsalicylic acid*	75 mg
	*Hydrochlorothiazide*	25 mg
	*Lovastatin*	40 mg
Diuretic drug + β-blocker + Statin (DiuBetSta)	*Hydrochlorothiazide*	25 mg
	*Atenolol*	50 mg
	*Lovastatin*	40 mg
Aspirin + β-blocker + Statin (AsaBetSta)	*Acetylsalicylic acid*	75 mg
	*Atenolol*	50 mg
	*Lovastatin*	40 mg
Aspirin + Diuretic drug + β-blocker + Statin (AsaDiuBetSta)	*Acetylsalicylic acid*	75 mg
	*Hydrochlorothiazide*	25 mg
	*Atenolol*	50 mg
	*Lovastatin*	40 mg
Hypothetical polypill	*Acetylsalicylic acid*	75 mg
	*Hydrochlorothiazide*	12.5 mg
	*Atenolol*	25 mg
	*Nifedepine*	20 mg
	*Lovastatin*	20 mg
	*Folic acid*	1 mg

ACE-inhibitors and other more recent drug classes are not included in the analysis because they are too expensive for widespread use in Africa and would most likely be dominated within our analytical framework. Whether or not these recent drugs are good choices for patients with multiple contraindications or more complex risk patterns (e.g. diabetes) would have to be assessed in a separate analysis.

We assume that cardiovascular risk can be assessed at regular health check-ups by considering risk factors that are easily observable (age, blood pressure, sex, etc.) [14]. It is assumed that two annual outpatient visits for monitoring treatment and risk factors are necessary for low and medium risk patients. For patients with high and very high risk it is assumed that four visits are necessary the first year and two annual visits thereafter. Finally, we assume that such preventive cardiology can be performed within the existing health service delivery system, but that some resources are necessary for public awareness campaigns and training of medical personnel.

## Methods

### Markov process model

We use a Markov process model to calculate clinical outcomes and costs during the life cycles of people under the alternative treatment scenarios. We assume that people are eligible for preventive cardiology at the age of 45, and that thereafter they receive treatment for their remaining life spans. There are two exceptions to the latter assumption. First, since our analysis is restricted to primary prevention, only the first event is registered, and people who experience a non-fatal stroke or coronary heart disease (CHD) are transferred to secondary prevention. Second, we assume that the provision of statins is stopped at the age of 80, since the effect of such treatment is not well documented for older people [18].

**Figure 1:**
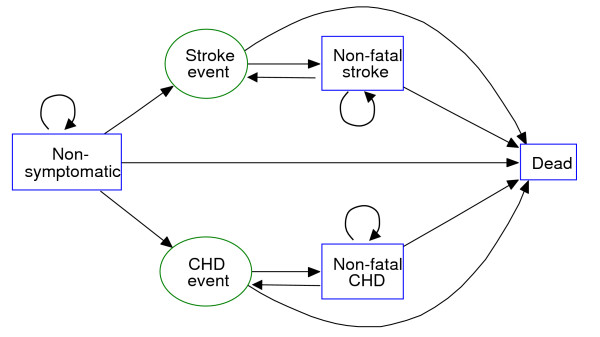
The life cycle model used to calculate the costs-, effects and cost-effectiveness of the alternative interventions.

In the model (Figure 1), all people start out as non-symptomatic. For each year that passes (Markov stage) people may remain well, they may experience stroke or CHD, or they may die from other causes not related to preventive cardiology. The probabilities for each of these outcomes depend on the individual risk profiles, people's age and the drugs being offered (see details below). The assumed case fatality rates for Stroke and CHD are 27% and 51%, respectively [19]. As mentioned earlier, people who experience non-fatal CV attacks are transferred to secondary prevention. In other words we assume that they are removed from preventive therapy, but the remaining life expectancies are nevertheless calculated (without the beneficial effects of preventive cardiology).

We used disability adjusted life years (DALYs) as the measure of clinical outcome. DALYs were calculated using standard assumptions [4], and a Tanzanian life table with a life expectancy at birth of 46.5 years [20] was used to assess the all cause risk of death at different ages (Table 2). For the non-fatal cases, disability weights of 0.49 and 0.268 are applied for CHD and stroke, respectively [4]. All monetary values are presented in 2005 US dollars (USD) and costs and consequences are discounted at 3% at baseline.

**Table 2:** Example of index patients, with annual risks of stroke or CHD events, and all cause risk of death.

**Index patient**	***Very high***	***High***	***Medium***	***Low***	***Very high***	***High***	***Medium***	***Low***	
**Sex**	Male	Male	Male	Female	Male	Male	Male	Female	
**SBP**	170	160	150	150	170	160	150	150	
**Smoking**	Yes	No	No	No	Yes	No	No	No	

**Age**	**Annual risk of stroke**				**Annual risk of CHD**				**All cause mortality**

**42**	0.006	0.003	0.003	0.001	0.015	0.008	0.007	0.004	0.019
**47**	0.007	0.004	0.003	0.001	0.019	0.011	0.010	0.007	0.020
**52**	0.009	0.005	0.004	0.002	0.023	0.014	0.013	0.009	0.021
**57**	0.011	0.006	0.005	0.003	0.027	0.017	0.016	0.011	0.024
**62**	0.014	0.008	0.007	0.004	0.031	0.021	0.019	0.012	0.032
**67**	0.018	0.010	0.009	0.005	0.035	0.024	0.022	0.013	0.043
**72**	0.023	0.012	0.011	0.007	0.038	0.027	0.025	0.013	0.062

### Calculating absolute risk

We calculated cost-effectiveness for four different risk levels of cardiovascular events; low, medium, high and very high; using an absolute risk approach. This implies that the risk of cardiovascular disease for an individual is assessed by taking into account all her or his known determinants of risk rather than thresholds for single risk factors [12,21]. Absolute cardiovascular risk can be assessed relatively easily in a clinical setting using standard methodology, although there exist no risk-instrument validated for use in our context [10].

For the purpose of this study, we constructed four index patients labeled "very high", "high", "medium" and "low" using algorithms from the Framingham study [22,23]. The index patients were stratified by varying, blood pressure (SBP), sex and smoking status, such that the annual total CV risk for a person aged 50 with a very high risk profile is higher than 3%. The definitions of people with high, medium and low CV risk were set at 2–3%, 1.5–2% and < 1.5%, respectively. The Framingham equations were also used to calculate the impact of age on the risk of stroke and CHD, as reported in Table 2.

In the risk calculations we used Tanzanian data on diabetes prevalence [24], but assumed that patients otherwise are asymptomatic, have no history of cardiovascular events, and that they are not on hypertension treatment when entered into the model. The remaining variables in the Framingham equations are kept constant at mean observed values from Framingham [22,23].

The above method only describes index-patients as examples of people with different risk levels. In our main analysis we calculate the effect of interventions for given absolute levels of risk. The above assumptions are therefore not critical to the main analysis as long as the findings and conclusions of the analysis are interpreted accordingly, In the clinical setting, however, CV risk must be assessed for individual patients and the choice of risk instruments then becomes crucial.

### Calculating treatment effects

Treatment effects for low dose diuretics and β-blockers, acetylsalicylic acid and statins used as monotherapy were taken from recent systematic reviews and meta-analyses from Europe and USA [17,25]. Unfortunately, we found no large long term clinical trials from Africa of drugs to prevent CVD. Nor did we find any meta-analyses reporting the effect of calcium antagonist compared to placebo, so we used a study reporting the relative risk compared to diuretics and β-blockers [26].

For drug combinations we assumed multiplicative effects [12,17]. This implies that the relative risk of the second drug in e.g. a duo-therapy applies to the remaining risk after the risk reduction of the first drug has been calculated. The assumption of multiplicative effects does not seem to be controversial, as the drugs influence different physiological mechanisms. It is potentially more problematic that studies on treatment effects generally have been performed in Caucasian populations, and the transferability to black African populations is uncertain. The applied treatment effects for the different drugs are reported in Table 3.

**Table 3:** Treatment effects (relative risks) with 95% confidence intervals (CI) of the alternative drugs.

**Drug**	**RR Stroke (95% CI)**	**RR CHD (95% CI)**	**Ref.:**
Acetylsalicylic acid (Asa)	0.84 (0.75 – 0.93)	0.68 (0.60 – 0.77)	[17]
Diuretic drug (Diu)	0.66 (0.55 – 0.78)	0.72 (0.61 – 0.85)	[25]
β-blocker (Bet)	0.71 (0.59 – 0.86)	0.93 (0.80 – 1.09)	[25]
Calcium antagonist vs diuretic drug or β-blocker (Cab)	0.87 (0.77 – 0.98)	1.12 (1.00 – 1.26)	[25, 26]
Statin (Sta)	0.83 (0.75 – 0.91)	0.39 (0.29 – 0.49)	[17]
Hypothetical polypill	0.20 (0.13 – 0.29)	0.12 (0.09 – 0.16)	[17]

### Calculating cost

We calculated drug costs based on dosages from standard treatment guidelines and information from the International Drug Price Indicator Guide [27]. We used the median prices of the available buyer prices, representing so called c.i.f. (cost free on board + insurance + freight). In order to approximate the c.i.f.-s to local opportunity costs, they were adjusted with a so called domestic margin of 1.43. This adjustment has been described elsewhere [13]. The prices of combination therapies were calculated by adding the costs of the individual ingredients. The applied prices of the different drugs are given in Table 4.

**Table 4:** Societal prices including markups of the alternative drugs. Minimum and Maximum values are calculated as most likely value -/+ 20%, respectively.

**Intervention**	**Strength/tablet**	**Price/tablet (USD) Source: [27]**	**Drug cost per year (USD)**	**Min**	**Max**
Acetylsalicylic acid (Asa)	75 mg	0.0118	6.16	4.93	7.39
Hydrochlorothiazide (Diu)	25 mg	0.0030	1.57	1.25	1.88
Atenolol (Bet)	50 mg	0.0084	4.38	3.51	5.26
Nifedepine (Cab)	20 mg	0.0281	29.33	23.47	35.20
Lovastatin (Sta)	20 mg	0.0520	54.28	43.43	65.14
Folic acid	1 mg	0.0271	14.14	11.32	16.97

Facility costs are based on a comprehensive costing study of Tanzanian health facilities [28]. To calculate the most likely cost per health check up, we used the mean cost of outpatient visits at four different health centers in Tanzania. The recurrent facility costs of 0.90 USD per outpatient visit include staff, medical supplies, utilities, stationary, uniforms and linen, cleaning, maintenance and travel [28]. Minimum and maximum values for uncertainty analysis were set at 0.70 and 1.22 USD per outpatient visit. Capital facility costs of 0.32 USD per visit include annualized costs of buildings, equipment, furniture and transportation [28]. Minimum and maximum capital costs are set at 0.13 and 0.57 USD per visit. We standardize the discount rate used in the costing study from 10% to 3%.

Well trained physicians can relatively easily assess cardiovascular risk of their patients at regular health check-ups or when they see patients for other reasons. However, although such passive case detection is certainly possible, we acknowledge that widespread implementation of a program of preventive cardiology warrants resources for awareness campaigns, training of physicians and administration above the facility level. We therefore apply a fixed ratio of 20% of facility costs to cater for program costs of scaling up preventive cardiology.

We did not include indirect patient costs (e.g. productivity changes) or saved treatment costs from averted cases of stroke and CHD. Although treatment costs in many settings are likely to be substantial, it remains to be established that good quality treatment and rehabilitation of CV patients is currently a widespread practice in the Tanzanian health care system.

### Net health benefits

We apply a net health benefit (NB) approach that disentangles the cost-effectiveness assessment from potential problems with ratio statistics as well as with interpretation of the findings [29,30] by using the formula below [31,32]:

*NB*_*i *_= Δ*DALY*_*i *_- (Δ*Cost*_*i*_/*λ*)

The above formula is based on a simple rearrangement of the decision rule that interventions are considered cost-effective if the incremental cost-effectiveness ratio is smaller than some cost-effectiveness threshold or value of ceiling ratio (λ), and by defining the net benefits as the balance on the right side of the intermediate expression [30]:

ΔCostiΔDALYi<λ ⇔ 0<ΔDALYi−ΔCostiλ⇒NB=ΔDALYi−ΔCostiλ
 MathType@MTEF@5@5@+=feaafiart1ev1aaatCvAUfKttLearuWrP9MDH5MBPbIqV92AaeXatLxBI9gBaebbnrfifHhDYfgasaacH8akY=wiFfYdH8Gipec8Eeeu0xXdbba9frFj0=OqFfea0dXdd9vqai=hGuQ8kuc9pgc9s8qqaq=dirpe0xb9q8qiLsFr0=vr0=vr0dc8meaabaqaciaacaGaaeqabaqabeGadaaakeaadaWcaaqaaiabfs5aejabdoeadjabd+gaVjabdohaZjabdsha0naaBaaaleaacqWGPbqAaeqaaaGcbaGaeuiLdqKaemiraqKaemyqaeKaemitaWKaemywaK1aaSbaaSqaaiabdMgaPbqabaaaaOGaeyipaWdcciGae83UdWMaeeiiaaIaeyi1HSTaeeiiaaIaeGimaaJaeyipaWJaeuiLdqKaemiraqKaemyqaeKaemitaWKaemywaK1aaSbaaSqaaiabdMgaPbqabaGccqGHsisldaWcaaqaaiabfs5aejabdoeadjabd+gaVjabdohaZjabdsha0naaBaaaleaacqWGPbqAaeqaaaGcbaGae83UdWgaaiabgkDiElabd6eaojabdkeacjabg2da9iabfs5aejabdseaejabdgeabjabdYeamjabdMfaznaaBaaaleaacqWGPbqAaeqaaOGaeyOeI0YaaSaaaeaacqqHuoarcqWGdbWqcqWGVbWBcqWGZbWCcqWG0baDdaWgaaWcbaGaemyAaKgabeaaaOqaaiab=T7aSbaaaaa@6F78@

The net-benefit formula compares the DALYs averted by an intervention under consideration (DALY_i_) with the minimum DALYs averted that would be necessary to consider an intervention cost-effective given a certain cost (Cost_i_). At the same time we get a linear expression rather than a ratio, thus avoiding several potentially problematic features of ratio statistics [29]. An intervention (i) is considered cost-effective if it produces a positive net benefit, in other words, if it averts more DALYs than the minimum implied by the threshold. A negative net benefit, on the other hand, means that the intervention is considered to be not cost-effective.

### Handling of uncertainty

We use distributions to capture the varying degree of inherent uncertainty in the variables. This probabilistic approach assesses simultaneous uncertainty in many variables and is well suited to express overall model uncertainty.

Monte Carlo simulations [33] were done with 5000 iterations. For each iteration a value is randomly drawn from each distribution and net health benefits calculated [32]. We assume that all relative risks are normally distributed, and calculate standard deviations (SD) based on confidence limits from the underlying meta-analyses [17,25,26]. For drug costs [27] we apply triangular distributions with minimum and maximum values at 20% below and above most likely values, respectively. A triangular distribution was chosen also for capital and recurrent facility costs, and maximum and minimum values were set as the lowest and highest values from the health centers in the costing study [28].

One-way sensitivity analyses are performed for key variables involving value choices. For the discount rate in DALY calculations we apply a range from 0% (no discounting) to 6%, with 3% as baseline. The age-weight modeling factor in the DALY calculation [4] is dichotomously varied from 1 (baseline) to 0 (no age-weighting).

### Cost-effectiveness acceptability curves

The results are presented as cost-effectiveness acceptability curves using standard methods [32,34]. Acceptability curves illustrate the probability that any particular intervention (i) is cost-effective or has a positive net health benefit, conditional on the willingness to pay per DALY (λ). For readers who are accustomed to traditional cost-effectiveness league tables, it is useful to note that the willingness to pay at 50% probability of being optimal for an intervention corresponds to the mean incremental cost-effectiveness ratio (ICER) of that intervention compared to the null-intervention (the alternative of doing none of the interventions). The curves also illustrate the degree of uncertainty in the estimates. Acceptability curves with steep slopes illustrate relatively certain outcomes, while curves that are less steep mean that the outcomes are relatively more uncertain.

Acceptability curves are used with increasing frequency in economic evaluations of health interventions, but have to our knowledge been applied only once before in a developing country setting [35]. All modeling and calculations were done using standard software [31].

### Cost-effectiveness frontiers

We also present the findings as cost-effectiveness acceptability frontiers that illustrate the probability of any intervention being optimal compared to all its alternatives. The optimal intervention is defined as the one with the highest expected net health benefit [32]. Cost-effectiveness frontiers also illustrate the crossover when one intervention is substituted by another as the one with the highest probability of being optimal, and therefore provide useful information for policy makers.

## Results

### Costs and effects of the interventions

The hypothetical Polypill yields 6.3 expected life years per person, which as expected is the highest of the alternatives since this is the most comprehensive treatment. The intervention yielding fewest life years is the β-blocker (Atenolol) given as monotherapy, with 0.6 expected life years per person. The life years saved per person are presented non-discounted and non-age weighted in Table 5.

**Table 5:** Mean life time health outcomes, costs and average and incremental cost-effectiveness ratios (CERs) for the drug combinations in a scenario with very high CV risk. Incremental CERs are in addition reported for the high, medium and low CV risk scenarios. 95% confidence intervals are reported in brackets for the ICERs.

	**Very high CV risk**					**Incremental CERs (USD/DALY)**		
**Intervention**	**Life years saved (nominal)**	**Costs**	**DALYs**	**ACER (USD/DALY)**	**ICER (USD/DALY)**	**High risk**	**Medium risk**	**Low risk**

**Asa**	1.6	138	0.8	163	(Dominated)			
**DiuVhigh**	1.6	74	0.9	85	85 (61 – 133)	135 (95 – 212)	149 (105 – 230)	232 (163 – 266)
**Bet**	0.6	107	0.3	329	(Dominated)			
**Cab**	0.7	444	0.4	1095	(Dominated)			
**Sta**	2.7	882	1.6	540	(Dominated)			
**AsaDiu**	3.1	175	1.6	111	143 (108 – 197)	222 (146 – 314)	242 (182 – 337)	377 (280 – 530)
**AsaBet**	2.2	208	1.1	182	(Dominated)			
**DiuBet**	2.2	141	1.1	124	(Dominated)			
**AsaDiuBet**	3.6	250	1.8	138	317 (169 – 3900)	532 (265 – *)	601 (305 – *)	1009 (465 – *)
**AsaDiuSta**	4.9	1123	2.6	431	(Dominated)			
**DiuBetSta**	4.6	1086	2.5	433	(Dominated)			
**AsaBetSta**	4.4	1143	2.4	481	(Dominated)			
**AsaDiuBetSta**	5.4	1229	2.8	440	999 (752 – 1376)	1588 (1175–2190)	1739 (1270–2420)	2749 (2010–3850)
**Poly**	6.3	1755	3.2	557	1476 (545–8000)	2466 (856–16800)	2735 (932–19500)	4589 (1450–51000)

* > 100 000

While the Polypill is the treatment yielding most life years of the alternatives, it is also the most expensive alternative. The expected lifetime cost of such treatment is USD 1755 per person, while the cheapest alternative is the diuretic drug (Hydrochlorothiazide), with an expected lifetime cost of USD 74 per person (Table 5).

### Cost-effectiveness

The favorable price of Hydrochlorothiazide (Diu) is the major reason for this intervention having the lowest average cost-effectiveness ratio (ACER) of the alternatives. When given as monotherapy to patients with very high CV risk this intervention cost 85 USD per DALY averted with baseline assumptions on discounting and age-weighting. This ratio is not significantly lower than when Hydrochlorothiazide is combined in duo-therapy with Aspirin or a β-blocker, in triple-therapy with Aspirin and a β-blocker, or than Aspirin given as mono-therapy. When Hydrochlorothiazide is combined with Aspirin, the ACER increases to 111 USD per DALY. The intervention with the least favorable ACER is the calcium antagonist (Nifedepine) given to people with low CV risk, with an ACER of USD 1095 per DALY averted. The mean average cost-effectiveness ratios (ACERs) for the 14 drugs in the scenarios of very high risk relative to the null-intervention are given in Table 5.

While ACERs are useful background information, rank ordering of interventions must be done on the basis of incremental cost-effectiveness ratios (ICERs) after removal of dominated interventions [33]. From Table 5 it can be seen that in the very high risk scenario the ICERs increase from 85 USD per DALY for Hydrochlorothiazide, to 143, 317, 999 and 1476 USD per DALY when Hydrochlorothiazide is combined with Aspirin, Aspirin and a β-blocker (Atenolol), Aspirin a β-blocker and a statin (Lovastatin), and the Polypill, respectively. All the other interventions are dominated either by strong or extended dominance.

Table 5 also includes the ICERs for the scenarios of high, medium and low CV risk, which are higher because the absolute CV risk and thus the absolute risk reduction is smaller for these groups.

### Uncertainty and value choices

**Figure 2:**
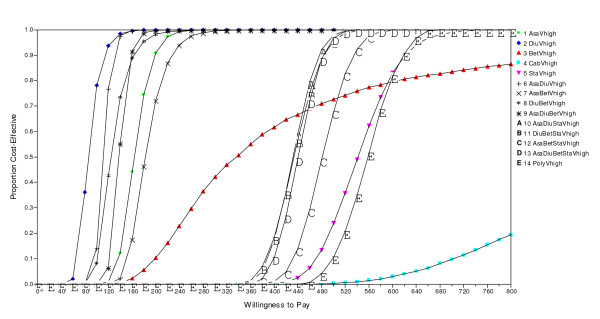
Cost-effectiveness acceptability curves for the alternative interventions in the scenario with very high CV risk.

The uncertainty surrounding the above mean ICERs is captured by the cost-effectiveness acceptability curves in Figure 2. Some interventions have steep acceptability curves, illustrating a relatively high level of certainty. The 95% confidence interval (CI) for the ICER of Hydrochlorothiazide given to people with very high CV risk, for example, is 61 – 133 USD per DALY. Hydrochlorothiazide in combination with Aspirin have a CI of 108–197 USD per DALY relative to Hydrochlorothiazide as monotherapy.

Interventions including Atenolol or Nifedepine, on the other hand, have acceptability curves that are fare less steep, illustrating higher levels of model uncertainty. The ICER of Atenolol in combination with Aspirin and Hydrochlorothiazide relative to the previous intervention, for example, has a CI of 169–3900 USD per DALY. The mean ICERs with CIs for the non-dominated interventions are given in Table 5. Figure 2 illustrate the acceptability curves for people with very high risk. For lower levels of CV risk, the acceptability curves for all interventions shift rightwards, while their shapes are roughly the same.

Discounting of future health benefits and age-weighting are controversial elements of the DALY methodology. One-way sensitivity analysis for example for Hydrochlorothiazide reveals that when the discount rate on health effects is set to 0% (no discounting) the ICERs improve from 85 at baseline (3%) to 70 USD per DALY. When the discount rate is increased from 3% to 5%, the ICER increase to 101 USD per DALY and become less attractive in terms of cost-effectiveness.

Similarly, when the DALYs are calculated without age-weights [4] the ICERs improve, in the case of Hydrochlorothiazide from 85 to 67 USD per DALY. This happens because the age weights give less weight to the life years earned by the elderly, who are the primary targets of preventive cardiology. Neither age-weighting nor discounting affect the relative ranking of the interventions in this study since all interventions target the same age-groups within similar time spans. But it is clear that the use of age-weighted and discounted DALYs disfavors preventive cardiology compared to e.g. interventions targeting childhood diseases.

### Which interventions are the best choices?

**Figure 3:**
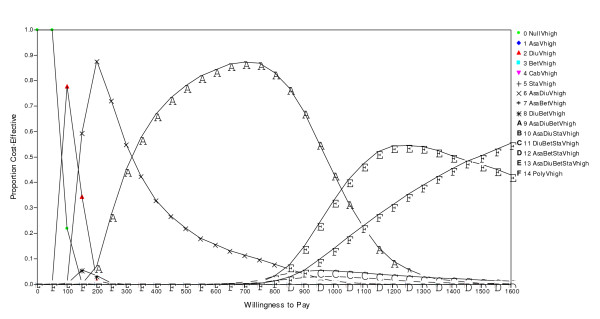
Cost-effectiveness acceptability frontiers in the scenario with very high CV risk.

None of the alternative drugs appear to be cost-effective if the societal willingness to pay for health is lower than 85 USD per DALY. If the willingness to pay is in the range 85–143 USD per DALY, Hydrochlorothiazide as monotherapy turn out as the optimal choice for people with very high risk (>3% annual risk) of cardiovascular disease. For the range 143 – 317 USD per DALY duo-therapy with Acetylsalicylic acid and Hydrochlorothiazide is the optimal choice to very high risk people, while a triple therapy with Acetylsalicylic acid, Hydrochlorothiazide and Atenolol turns out to be optimal in the range 317 – 999 USD per DALY. Finally, when the societal willingness to pay for health is in the ranges 999–1476 or higher than 1476 USD per DALY, the optimal strategies are to treat patients with very high risk with a combination of Acetylsalicylic acid, Hydrochlorothiazide, Atenolol and Lovastatin and the hypothetical Polypill, respectively. All the other treatment strategies are dominated by the above mentioned. The above cut-off points are illustrated in the cost-effectiveness acceptability frontier in Figure 3, along with the probabilities that either policy will maximize the averted DALYs for different levels of willingness to pay for health.

The main trend when we change the focus from patients with very high CV risk to lower risk groups is that the cut-off points between optimal strategies are increased as the level of risk is reduced (Figure 3). The null intervention, for example, is probably optimal until the societal willingness to pay for health exceeds 135 USD per DALY in the high risk group, while the cut-offs are 149 and 232 in the medium and low risk scenarios, respectively.

**Figure 4:**
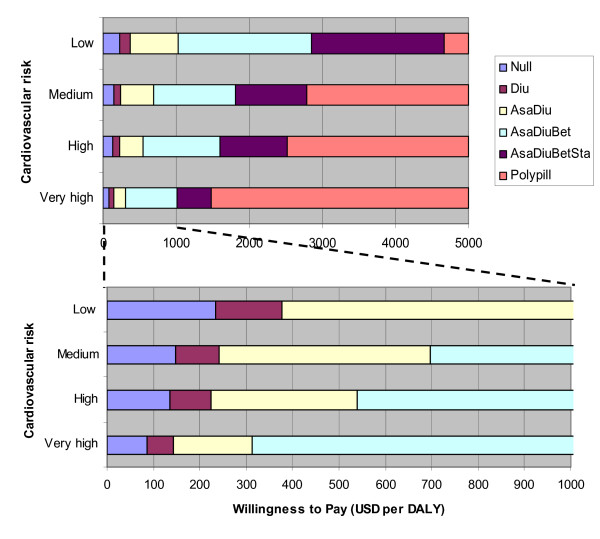
The optimal strategies (yielding max net health benefits) under different risk scenarios for different levels of societal willingness to pay for health. Scales USD 0–5000 per DALY (above) and USD 0–1000 per DALY (below).

Figure 4 shows that if decision makers in countries such as Tanzanian have a willingness to pay for health of for example 50 USD per DALY, none of the treatment alternatives to any of the risk groups should be publicly financed from a health maximization point of view. If the willingness to pay is somewhat higher, for example 100 USD per DALY, the optimal treatment mix is to provide Hydrochlorothiazide (Diu) to people with very high CV risk, but no treatment to other risk groups. For a willingness to pay of say 200 USD per DALY, the optimal policy mix would be to provide duotherapy with Acetylsalicylic acid and Hydrochlorothiazide (AsaDiu) to people with very high risk, monotherapy with Hydrochlorothiazide (Diu) to patients with medium and high risk, and no drugs to people in the low risk group.

## Discussion

Our study suggests that the use of Hydrochlorothiazide to people with high to very high cardiovascular risk is likely to be the most favorable choice of intervention when the willingness to pay is higher than some 88 USD per DALY. Hydrochlorothiazide and several of the alternative drug combinations costs less than Gross Domestic Product (USD 300) per capita per DALY averted, and may therefore be classified as *very cost-effective *according to the Commission on Macroeconomics and Health [36].

Our findings render preventive cardiology somewhat less attractive in terms of cost-effectiveness compared to the findings of Murray and colleagues. They report CERs for an absolute risk approach with statin, diuretic, β-blocker and aspirin, which is comparable to the AsaDiuBetSta intervention in our study, in the range 230 – 1010 international dollars per DALY depending on absolute risk level. When these findings are converted to USD using a purchasing power parity of 357 [37] and exchange rate for year 2000 of 800 TSh/USD, respectively, the ACERs for these alternatives become 103 – 454 USD per DALY. In comparison, our study finds a range for the ACERs of 440 – 1148 for these interventions (Table 5). Different analytical models may explain some of this disparity, but also the fact that we apply a Tanzanian life table with a life expectancy at birth of only 46 years is likely to explain why our model seems to produce fewer DALYs than Murray and colleagues' study. The differences are not sufficiently large to change the conclusion in both papers that preventive cardiology is cost-effective based on standard definitions.

A country like Tanzania has a public health expenditure of only 6 USD per capita per year [38], which is typical for many countries in sub-Saharan Africa. The above conclusion that some of the interventions are cost-effective should therefore be done with caution and does not automatically imply than any of the interventions should be recommended for implementation. First, our findings should be interpreted within the framework of a larger decision problem and compared with alternative health interventions with low coverage. Obviously, it is necessary to consider potential biases caused by differences in perspectives and methodology when making such comparisons. It has been demonstrated that prevention of mother to child transmission of HIV is more cost-effective than any of the treatment alternatives in our analysis [39,40]. Other examples of interventions with low coverage and ICER appearing to be more favorable in terms of health maximization than preventive cardiology are improved case management of acute childhood diarrhea [41], insecticide treated bednets or intermittent presumptive treatment in pregnant women to prevent malaria [42].

Second, our index patients should be interpreted as examples of very high, high, medium and low cardiovascular risk, respectively, and whether or not the Framingham population resembles a Tanzanian population is therefore not necessarily a critical assumption. There are few studies from the region on cardiovascular risk, and there is no evidence on outcomes from intervention studies in sub-Saharan Africa. The possibility that our effect-estimates are biased therefore cannot be ruled out. It has been suggested that stroke is relatively more important than CHD in African compared to western Caucasian populations, particularly because blacks in Africa generally have lower serum cholesterol levels and higher HDL-levels [11,43], while other evidence suggest that the contributions of stroke and CHD to burden of disease in Tanzania are quite similar [44]. If stroke indeed is the most important in Tanzania, the cost-effectiveness of drug combinations including Acetylsalicylic acid and statin, that have their primary effect on CHD, may have been too favorably estimated in our analysis, while there might be a bias against interventions controlling blood pressure, that are relatively more effective in prevention of stroke. Moreover, side-effects of monotherapy with Acetylsalicylic acid has been suggested to be more problematic in this setting, and treatment with Acetylsalicylic presuppose adequate blood pressure control [45].

Third, the efficacy estimates include hard end points (mortality), and adverse treatment effects with fatal outcomes are therefore included in our analysis. However, non-fatal adverse treatment effects, like non-fatal bleeding caused by acetylsalicylic acid, are not modeled. The excess absolute risk for major non-fatal bleeds, however, is only 0.4 [17], which in our models is unlikely to significantly affect the findings.

We have not calculated the budget impact of a widespread adoption of the interventions in this paper. The incremental drug costs per year of treatment are reported in Table 4, and the life time costs per patient are reported in Table 5, but since the prevalence of different risk levels is not known in Tanzania, the costs of scaling up the programs are difficult to calculate accurately. The budget impact is, however, likely to be large because a large fraction of the population probably would be eligible for treatment [7, 46].

Finally, we have considered preventive cardiology from an efficiency point of view, where the objective is to maximize societal health given the available resources. We believe that additional principles for allocation of scarce resources, like concern for the like concern for the distribution of DALYs, also need to be considered in a priority setting process. Although we have not made a formal analysis of this, it is not likely to give high weight to preventive cardiology since such treatment in essence is about treating people of relatively high age that are not yet sick.

## Conclusion

Many countries in sub-Saharan Africa are facing high and rising levels of cardiovascular disease, and are currently considering how to meet this challenge. A range of pharmacological interventions for preventive cardiology are *very cost-effective *in a sub-Saharan African setting like Tanzania, with Hydrochlorothiazide to patients with high to very high cardiovascular risk as one of the most likely candidates. Despite the attractiveness of this and other interventions in terms of cost-effectiveness, it is uncertain whether they should be implemented in a country like Tanzania. There are several other interventions with low coverage rates, e.g. treatment of childhood diarrhoea and malaria, that are even more cost-effective and that are competing for the same resources. Only when the societal willingness to pay or the alternative value of incremental budgets exceeds 85 USD per DALY, preventive cardiology is likely to be a good choice of policy in Tanzania.

One of the more sophisticated treatment alternatives, the Polypill, does at present not seem to be a relevant option for public funding in a country like Tanzania. But countries with more well-funded health systems, or private persons at risk who can afford to pay for the drugs themselves, would probably consider such treatment good value for money. This study suggests that monotherapies with Atenolol or Nifedepine are the weakest candidates for implementation among those compared.

## Competing interests

The author(s) declare that they have no competing interests.

## Authors' contributions

BR designed the analytical model, carried out the hands-on modeling in TreeAge, did the costing analysis and drafted the first version of the paper including figures and tables. OFN was main responsible for the review of literature on intervention effectiveness, the review of international prevention guidelines, contributed in the analytical design of the study and contributed in the drafting of the manuscript. YH assessed the Tanzanian applicability of the interventions, contributed with the interpretation of local prevention guidelines, and participated in the revision of the manuscript. All authors read and approved the final manuscript.
